# Correction: Woo et al. The Fate of Antibiotic Impregnated Cement Space in Treatment for Forefoot Osteomyelitis. *J. Clin. Med.* 2022, *11*, 1976

**DOI:** 10.3390/jcm11113237

**Published:** 2022-06-06

**Authors:** Inha Woo, Jeongjin Park, Hyungyu Seok, Tae-gon Kim, Jun Sung Moon, Seung Min Chung, Chul Hyun Park

**Affiliations:** 1 Department of Orthopedics, Yeungnam University Hospital, Daegu 38541, Korea; buonggiorno39@gmail.com (I.W.); wjdwls3912@naver.com (J.P.); rkaldhkthfl4@naver.com (H.S.); 2 Department of Plastic Surgery, College of Medicine, Yeungnam University, Daegu 38541, Korea; kimtg0919@daum.net; 3 Department of Internal Medicine, Division of Endocrinology and Metabolism, College of Medicine, Yeungnam University, Daegu 38541, Korea; mjs@yu.ac.kr (J.S.M.); smchung@ynu.ac.kr (S.M.C.); 4 Department of Orthopaedic Surgery, College of Medicine, Yeungnam University, Daegu 38541, Korea

## Additional Affiliations

In the published publication [1], there were not added the affiliations for new added authors Tae-gon Kim, Jun Sung Moon and Seung Min Chung. The newly added affiliations appear here.

Affiliation 2. Department of Plastic Surgery, College of Medicine, Yeungnam University, Daegu 38541, Korea

Affiliation 3. Department of Internal Medicine, Division of Endocrinology and Metabolism, College of Medicine, Yeungnam University, Daegu 38541, Korea

## Addition of co-Authors

Tae-gon Kim, Jun Sung Moon and Seung Min Chung were not included as authors in the original publication. The corrected Author Contributions Statement appears here.

**Author Contributions:** Conceptualization, C.H.P. and I.W.; data curation, H.S. and J.P.; methodology, T.-g.K., J.S.M. and S.M.C.; investigation, T.-g.K.; writing—original draft preparation, I.W.; writing—review and editing, H.S.; supervision, C.H.P. All authors have read and agreed to the published version of the manuscript.

The authors apologize for any inconvenience caused state that the scientific conclusions are unaffected. The original publication has also been updated.
